# Alcohol Consumption Per Capita and Suicide: A Meta-Analysis

**DOI:** 10.1001/jamanetworkopen.2025.33129

**Published:** 2025-09-22

**Authors:** Katherine Guo, Huan Jiang, Kevin D. Shield, Sheryl Spithoff, Shannon Lange

**Affiliations:** 1 Institute for Mental Health Policy Research, Centre for Addiction and Mental Health, Toronto, Ontario, Canada; 2 Institute of Medical Science, University of Toronto, Toronto, Ontario, Canada; 3 Dalla Lana School of Public Health, University of Toronto, Toronto, Ontario, Canada; 4 Women’s College Research Institute, Toronto, Ontario, Canada; 5 Department of Family and Community Medicine, University of Toronto, Toronto, Ontario, Canada; 6 Campbell Family Mental Health Research Institute, Centre for Addiction and Mental Health, Toronto, Ontario, Canada; 7 Department of Psychiatry, University of Toronto, Toronto, Ontario, Canada

## Abstract

**Importance:**

At the individual level, alcohol use is an established risk factor for suicide; however, it is unclear whether this is reflected at the population level. If alcohol consumption per capita (APC), a population-level metric of total alcohol consumption used in international frameworks to measure progress in reducing the harmful use of alcohol, is associated with suicide, it could prove to be a useful target for suicide prevention initiatives.

**Objective:**

To examine whether there is an association between APC and suicide mortality, and if there is, to evaluate whether it differs by sex.

**Data Sources:**

Embase, Medline, PsycINFO, and Web of Science were searched from database inception to February 24, 2025, for original quantitative studies that measured the association between APC and suicide.

**Study Selection:**

Included studies consisted of (1) original quantitative studies with a longitudinal observational or cross-sectional ecological design, including pre-post designs; and (2) studies that provided a measure of association. A total of 304 records were initially identified.

**Data Extraction and Synthesis:**

Data extraction was completed by 1 reviewer and cross-checked by a second review. Risk of bias was assessed using the Risk of Bias in Nonrandomized Studies of Exposure tool, and evidence quality was assessed using Grading of Recommendations, Assessment, Development, and Evaluations. The Preferred Reporting Items for Systematic Reviews and Meta-Analyses reporting guideline was followed. A random-effects meta-analysis was conducted to obtain a pooled estimate of the association between APC and suicide mortality. The presence of a sex difference was assessed using a random-effects meta-regression.

**Main Outcomes and Measures:**

The association between APC, measured as alcohol consumed in liters per person, and the suicide mortality rate.

**Results:**

A total of 13 studies were included in the main analysis. It was found that, on the population level, every 1-L increase in APC was associated with an increase of 3.59% (95% CI, 2.38%-4.79%) in the suicide mortality rate. There was no evidence of a sex difference in the association of interest.

**Conclusions and Relevance:**

In this systematic review and meta-analysis, an increase in APC was associated with an increase in the suicide mortality rate at the population level and that the association was similar across sexes. As such, APC may be a useful target to consider within comprehensive national suicide prevention strategies.

## Key Points

**Question:**

Is alcohol consumption per capita associated with suicide mortality and, if so, does the association differ by sex?

**Findings:**

This meta-analysis included 13 studies and found that a 1-L increase in alcohol consumption per capita was associated with a 3.59% increase in the suicide mortality rate. There was no evidence of a sex difference in this association.

**Meaning:**

These findings suggest that alcohol consumption per capita may be a useful target to consider within comprehensive national suicide prevention strategies.

## Teaser

This meta-analysis examines whether there is an association between alcohol consumption per capita and the suicide rate and, if so, whether this association varies by sex.

## Introduction

Suicide is a complex public health problem that substantially impacts global health. The World Health Organization estimated that in 2019 more than 700 000 people died by suicide around the world.^1^ Certain demographic characteristics may be associated with higher rates of suicide mortality, with sex differences at the individual level being well-documented.^2^ Suicide is largely preventable through a combination of individual- and population-level interventions,^1,3,4^ and the need to address suicide has been garnering increased attention from international public health agencies. In fact, suicide serves as the only mental health indicator in the United Nations (UN) Sustainable Development Goals (Target 3.4).^5^

Alcohol use is a well-established risk factor for suicide,^6,7^ with both acute and long-term effects at the individual level.^8,9,10^ Sex differences in alcohol consumption have also been observed, with male individuals typically demonstrating higher rates and levels compared with female individuals.^11,12^ The association between alcohol use and suicide is also strongly affected by sociocultural factors, which may be reflected in country-specific differences of the observed risk association.^13^

Implementation and monitoring of population-level public health interventions often rely on population-level health indicators, which provide quantifiable measures of health status and outcomes within populations. Thus, given the association between alcohol use and suicide on the individual level, population-level measures of alcohol consumption may be useful indicators to consider within national suicide prevention strategies. Population-level alcohol consumption is typically measured using sales and taxation data in lieu of self-reported survey data, as self-reported data may underreport the true level of consumption within a population by as much as 50%.^14,15,16^ These proxy measures are frequently denoted as alcohol consumption per capita (APC). APC has also been used to set international goals related to the reduction of alcohol-related harms, as seen within the UN Sustainable Development Goals (Target 3.5).^5^ While population-level interventions targeting alcohol consumption, such as alcohol taxation, affect consumption at the individual level, the impact of these interventions are best measured using population-level indicators, such as APC due to feasibility and limitations of individual-level consumption data. However, APC is a measure of total alcohol consumption in a population and is not a direct measure of drinking among drinkers or subpopulations (eg, individuals with depression) or a direct measure of drinking patterns.^17^ Thus, APC may or may not be a useful metric to consider as an indicator of progress with respect to suicide mortality.

Before we can draw any such conclusions, the risk association between APC and suicide mortality rates should be established. Existing literature reviews examining the relationship in question are either outdated^18,19,20^ and/or have a limited geographic scope,^21,22,23,24^ and to our knowledge, no meta-analysis currently exists for this association. Additionally, existing studies do not typically conduct formal tests for sex differences, instead relying on a theoretical basis to justify any observed differences. Therefore, the objective of the current study was 2-fold: (1) determine whether there is an association between APC and suicide mortality and (2) if there is a relationship, determine whether it differs by sex.

## Methods

The current systematic review and meta-analysis was reported according to the Preferred Reporting Items for Systematic Reviews and Meta-Analyses (PRISMA) 2020 statement.^25^ The protocol was registered with the International Prospective Register of Systematic Reviews (PROSPERO, registration number CRD42025643298).

### Search Strategy

A systematic search was conducted in Embase, Medline, PsycINFO, and Web of Science, spanning from database inception to February 24, 2025. A combination of MeSH terms and keywords for *alcohol consumption*, *population level*, and *suicide* were used (eAppendix 1 in Supplement 1). Manual reviews of citations in the articles deemed relevant and studies included in existing literature reviews were also conducted. The search results were imported into Covidence^26^ for deduplication.

### Study Selection and Data Extraction

Citations were imported into Covidence^26^ for title and abstract screening, and full-text review. Title and abstract screening was done by a single reviewer (K.G.). A second reviewer (S.L.) then cross-checked all excluded citations to identify potentially relevant studies that may have been wrongly excluded. These studies were then included for full-text review, which used a similar process. Data extraction was completed by 1 investigator (K.G.) using a template created in Excel version 2501 (Microsoft Corp). The template was initially piloted using 5 studies and revised to ensure all relevant data were extracted, then cross-checked by a second reviewer (S.L.).

### Eligibility Criteria

Studies had to (1) be original quantitative studies that used a longitudinal observational or cross-sectional ecological design, including pre-post designs; and (2) provide a measure of association. No restrictions were placed on geographical location or year of publication. No restriction was placed on language for the initial search; however, subsequent screening resulted in the exclusion of studies not written in English. Details pertaining to the initial inclusion and exclusion criteria are presented in Table 1.

**Table 1.** PICOS Criteria for Study Selection

PICOS criteria	Inclusion	Exclusion
Population	Population level	NA
Intervention or exposure	Alcohol consumption per capita measured using production, import, export, sales or taxation, and/or survey data	NA
Comparator	NA	NA
Outcome	Death by suicide (count or rate)	Aggregation of death by suicide and suicide attempt
Study design	Longitudinal observational study design or cross-sectional ecological study	NA
Other	Any language, geographic region or study year	Sample overlaps with another study that is more comprehensive or recent; dissertation or conference abstract

Abbreviations: NA, not applicable; PICOS, population, intervention or exposure, comparator, outcome, and study design.

To enhance the comparability of reported results, studies included in the meta-analysis had to (1) provide an effect estimate representing the percentage change in the suicide mortality rate associated with a unit-change in APC or an effect estimate that could be transformed to represent the respective measure of association as well as (2) a measure of error. For the formulas used, see eAppendix 2 in Supplement 1.

### Definitions

APC was defined as the total amount of alcohol consumed per person (age 15 years or older, unless otherwise specified) within a predefined time period, in liters of pure alcohol. Acceptable measures included production, import, export, sales or taxation, and/or survey data. Suicide was defined as death caused by self-directed injurious behavior with intent to die as a result of the behavior and/or the applicable *International Classification of Diseases* (*ICD*) codes (eg, *ICD-10* codes: X60-X84 and Y87.0).^27^ The suicide mortality rate was defined as the number of deaths by suicide in a predefined time period divided by the population and multiplied by 100 000. For the random-effects meta-regression, described later, geographical location was operationalized according to the World Health Organization (WHO) region in which the study took place, and study year was coded as the first year of the time-series or the year of the cross-sectional analysis.

### Statistical Analysis

A random-effects meta-analysis^28^ was performed to determine whether there is an association between APC and suicide mortality. For studies that provided comprehensive subgroup estimates (ie, provided sex- or beverage type–specific estimates) their estimates were first pooled using random-effects meta-analysis; the resulting estimate was subsequently included in the main meta-analysis. Heterogeneity was assessed using the *I*^2^ statistic.^29^ Publication bias was examined by visually inspecting the funnel plot.^30^ A random-effects meta-regression was performed to determine whether geographical location and study year significantly changed the association of interest. Studies that reported estimates pooled across multiple countries were not included in the respective meta-regression model. To determine whether the respective association differed by sex, a separate random-effects meta-regression was conducted on studies that provided sex-specific estimates. Statistical significance was set at *P* ≤ .05. All statistical analyses were conducted using R version 4.4.2 (R Project for Statistical Computing).^31^

#### Sensitivity Analyses

Two sensitivity analyses were performed to (1) examine the impact of including studies that only reported subgroup estimates that did not represent the overall general population (ie, studies that only reported a male-specific estimate), and (2) examine the impact of including potential outliers. Outliers were defined as any study reporting an effect estimate 3 or more SDs beyond the mean of all reported effect estimates.

#### Risk of Bias

Risk of bias was assessed by one reviewer (K.G.) using the “Risk of Bias in Nonrandomized Studies of Exposure” (ROBINS-E) tool for cohort studies.^32^ Each study was evaluated according to the ROBINS-E tool to assess for potential bias due to confounding, exposure measurement, participant selection, postexposure interventions, missing data, outcome measurement, and results reporting.

#### Grading of Recommendations, Assessment, Development, and Evaluations Assessment

Grading of Recommendations, Assessment, Development, and Evaluations (GRADE) was used to assess evidence quality according to the GRADE working group guidelines.^33^ Evidence quality was downgraded by 1 level for each major concern in the 5 domains: risk of bias, imprecision, indirectness, inconsistency, and publication bias.

## Results

**Figure 1.**
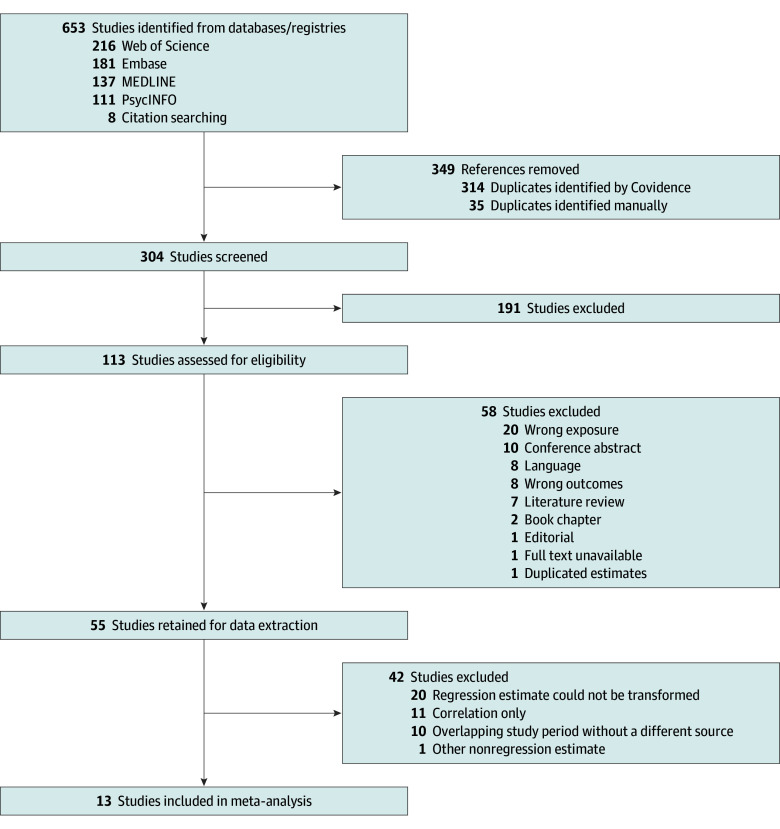
Study Flowchart

Following the removal of 349 duplicates, 304 records were screened using titles and abstracts; 113 studies subsequently underwent full-text review, and 55 studies were retained for data extraction. Of these 55 studies, a total of 13 studies^34,35,36,37,38,39,40,41,42,43,44,45,46^ were deemed eligible for inclusion in the meta-analysis (Figure 1).

An overview of the included studies, including the original and transformed effect estimates, is presented in Table 2. Individual estimates were available for a total of 23 countries. Two studies^36,38^ provided estimates pooled across multiple countries, collectively encompassing 78 different countries. Study periods ranged from 13 to 58 years (mean [SD], 38.56 [12.95] years). All studies used suicide mortality rate as their outcome.

**Table 2.** Characteristics of All Included Studies

Source	Study design	Country	Study period	Sex	Beverage type	Age, years	Model	Effect estimate (SE)	Transformed effect estimate (95% CI), %	Transformed SE
Norstrom,^40^ 1988	Longitudinal	Denmark	1931-1980	Both	NA	≥15	ARIMA	0.01 (0.03)	1.01 (−4.76 to 7.12)	3.03
		Finland	1932-1980					0.01 (0.2)	1.01 (−31.75 to 49.48)	20.20
		Norway	1931-1980					0.16 (0.04)	17.35 (8.50-26.92)	4.69
		Sweden	1922-1949					0.09 (0.04)	9.42 (1.17-18.34)	4.38
		Sweden	1950-1970					0.15 (0.04)	16.18 (7.42-25.66)	4.65
Caces et al,^42^ 1998	Longitudinal	United States	1934-1987	Both	NA	≥14	ARIMA	0.03 (0.02)	3.05 (−0.92 to 7.17)	2.06
				Male				0.039 (0.02)	3.98 (−0.02 to 8.13)	2.08
				Female				0.04 (0.02)	4.08 (0.08-8.24)	2.08
Chen et al,^36^ 2009	Longitudinal	OECD member countries (pooled)	1980-2003	Male	NA	≥15	Linear fixed effect	0.01 (0.02)	1.01 (−2.88 to 5.04)	2.02
				Female				0.02 (0.03)	2.02 (−3.81 to 8.20)	3.06
Landberg,^35^ 2008	Longitudinal	Russia	1959-1998	Both	NA	≥15	ARIMA	0.072 (0.009)	7.47 (5.59-9.38)	0.97
				Male				0.08 (0.01)	8.33 (6.23-10.47)	1.08
				Female				0.043 (0.006)	4.39 (3.17-5.63)	0.63
		Belarus	1970-2003	Both				0.055 (0.015)	5.65 (2.59-8.81)	1.58
		Poland	1959-1996	Both				0.066 (0.021)	6.82 (2.52-11.31)	2.24
				Male				0.074 (0.021)	7.68 (3.34-12.21)	2.26
				Female				0.015 (0.022)	1.51 (−2.77 to 5.98)	2.23
		Bulgaria	1964-2003	Both				0.045 (0.008)	4.60 (2.98-6.26)	0.84
				Male				0.043 (0.008)	4.39 (2.77-6.04)	0.84
				Female				0.059 (0.026)	6.08 (0.81-11.62)	2.76
		Czechoslovakia	1953-1991	Both				0.032 (0.015)	3.25 (0.26-6.33)	1.55
				Male				0.041 (0.015)	4.19 (1.17-7.29)	1.56
				Female				0.012 (0.022)	1.21 (−3.06 to 5.67)	2.23
		Hungary	1955-2002	Both				0.027 (0.013)	2.74 (0.15-5.39	1.34
				Male				0.031 (0.014)	3.15) (0.36-6.02)	1.44
				Female				0.017 (0.018)	1.71 (−1.81 to 5.37)	1.83
		Former German Democratic Republic	1960-1987	Both				0.031 (0.032)	3.15 (−3.12 to 9.83)	3.30
Mäkelä ,^43^ 1996	Longitudinal	Finland	1950-1991	Male	NA	≥15	ARIMA	0.04 (0.03)	4.08 (−1.86 to 10.38)	3.12
Norstrom,^41^ 1995	Longitudinal	France	1930-1987	Male	NA	≥15	ARIMA	0.027 (0.004)	2.74 (1.93-3.55)	0.41
		Sweden	1930-1987	Male	NA			0.099 (0.023)	10.41 (5.54-15.50)	2.54
Norstrom et al,^44^ 2018	Longitudinal	Sweden	1987-2015	Both	NA	≥15	Seasonal ARIMA	0.122 (0.026)	12.98 (7.36-18.88)	2.94
Norstrom et al,^45^ 2020	Longitudinal	Finland	1995-2016	Both	NA	≥15	Seasonal ARIMA	0.058 (0.009)	5.97 (4.12-7.86)	0.95
Norstrom et al,^34^ 2012	Longitudinal	Japan	1963-2007	Male	Beer	≥15	ARIMA	−0.07 (0.059)	−6.76 (−16.94 to 4.67)	5.50
				Male	Spirits			0.194 (0.083)	21.41 (3.18-42.86)	10.08
				Male	Wine			0.528 (0.472)	69.55 (−32.77 to 327.64)	80.03
				Male	Other alcohol			−0.058 (0.057)	−5.64 (−15.61 to 5.52)	5.38
				Female	Beer			−0.063 (0.066)	−6.11 (−17.50 to 6.86)	6.20
				Female	Spirits			0.096 (0.071)	10.08 (−4.22 to 26.51)	7.82
				Female	Wine			0.693 (0.462)	99.97 (−19.15 to 394.57)	92.39
				Female	Other alcohol			0.007 (0.051)	0.70 (−8.88 to 11.29)	5.14
Ramstedt,^46^ 2005	Longitudinal	Canada	1950-1998	Both	NA	≥15	ARIMA	0.042 (0.016)	4.29 (1.07-7.61)	1.67
				Male				0.038 (0.008)	3.87 (2.26-5.51)	0.83
				Female				0.066 (0.033)	6.82 (0.13-13.96)	3.53
Skog et al,^37^ 1995	Longitudinal	Portugal	1931-1989	Male	NA	Not specified	ARIMA	0.019 (0.006)	1.92 (0.73-3.12)	0.61
				Female				0.004 (0.009)	0.40 (−1.35 to 2.19)	0.90
Testa et al,^38^ 2019	Cross-sectional	76 Countries (pooled)	2010	Both	Spirits	≥15	Negative binomial (log-link)	0.156 (0.044)	16.88 (7.14-27.12)	5.10
					Wine			0.029 (0.032)	2.94 (−3.25 to 9.64)	3.29
					Beer			0.11 (0.042)	11.63 (−7.69 to 10.63)	4.67
					Other			−0.03 (0.070)	−2.96 (−15.38 to 11.18)	6.78
Ramstedt,^39^ 2001	Longitudinal	Austria	1950-1995	Male	NA	≥15	ARIMA	−0.015 (0.013)	−1.49 (−3.97 to 1.05)	1.28
				Female				0.009 (0.016)	0.90 (−2.21 to 4.12)	1.61
		Belgium		Male				0.045 (0.023)	4.60 (−0.01 to 9.43)	2.41
				Female				0.094 (0.032)	9.86 (3.18-16.97)	3.52
		Denmark		Male				−0.018 (0.029)	−1.78 (−7.21 to 3.96)	2.85
				Female				−0.002 (0.034)	−0.20 (−6.63 to 6.68)	3.39
		Finland		Male				0.036 (0.022)	3.67 (−0.71 to 8.23)	2.28
				Female				0.042 (0.028)	4.29 (−1.28 to 10.17)	2.92
		France		Male				0.002 (0.01)	0.20 (−1.74 to 2.18)	1.00
				Female				0.001 (0.013)	0.10 (−2.42 to 2.68)	1.30
		Ireland		Male				0.032 (0.05)	3.25 (−6.39 to 13.88)	5.16
				Female				0.011 (0.079)	1.11 (−13.40 to 18.04)	7.99
		Italy		Male				−0.013 (0.014)	−1.29 (−3.96 to 1.45)	1.38
				Female				0.003 (0.012)	0.30 (−2.03 to 2.69)	1.20
		Netherlands		Male				−0.016 (0.019)	−1.59 (−5.18 to 2.15)	1.87
				Female				0.065 (0.024)	6.72 (1.81-11.86)	2.56
		Norway		Male				0.121 (0.06)	12.86 (0.34-26.95)	6.77
				Female				0.22 (0.059)	24.61 (11.00-39.88)	7.35
		Portugal		Male				0.016 (0.005)	1.61 (0.62-2.61)	0.51
				Female				0.014 (0.009)	1.41 (−0.36 to 3.21)	0.91
		Spain		Male				−0.028 (0.012)	−2.76 (−5.02 to −0.45)	1.17
				Female				0.001 (0.014)	0.10 (−2.61 to 2.88)	1.40
		Sweden		Male				0.101 (0.03)	10.63 (4.31-17.33)	3.32
				Female				0.079 (0.035)	8.22 (1.05-15.90)	3.79
		United Kingdom		Male				−0.011 (0.023)	−1.09 (−5.45 to 3.47)	2.27
				Female				0.027 (0.032)	2.74 (−3.51 to 9.39	3.29
		West Germany		Male				0.006 (0.014)	0.60) (−2.12 to 3.40)	1.41
				Female				0.029 (0.015)	2.94 (−0.04 to 6.01)	1.54

Abbreviations: ARIMA, autoregressive integrated moving average; NA, not applicable; OECD, Organisation for Economic Co-operation and Development.

**Figure 2.**
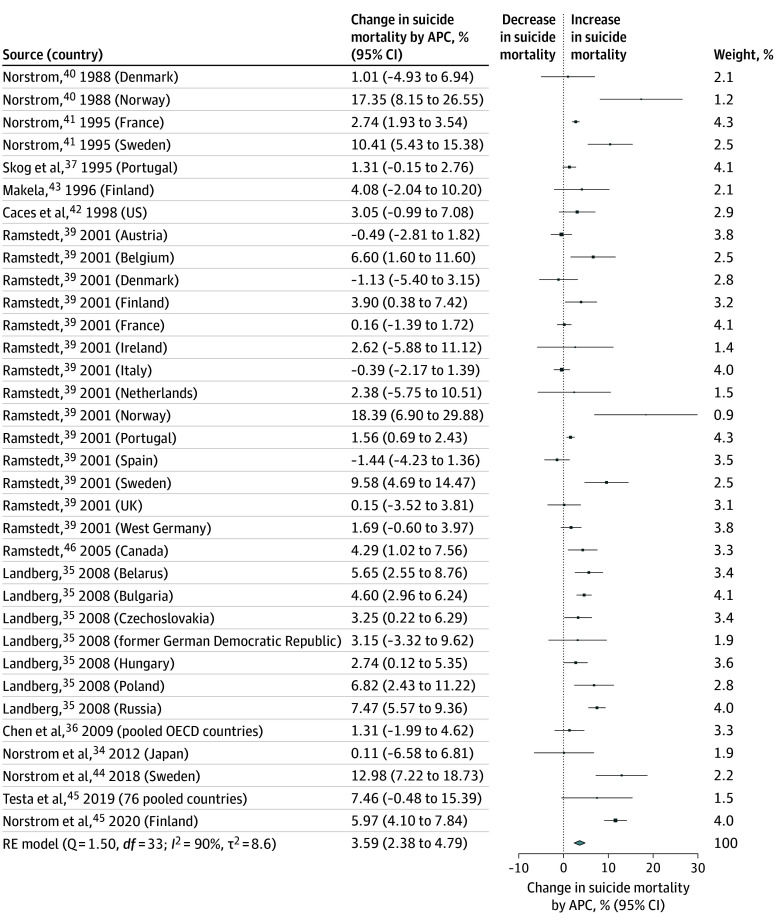
Association Between Alcohol Consumption Per Capita (APC) and Suicide Mortality Sizes of boxes indicate weight of each study in the model. OECD indicates Organisation for Economic Co-operation and Development.

The main meta-analysis included all 13 studies (representing 34 estimates) and found that there was a 3.59% (95% CI, 2.38%-4.79%) change in the suicide mortality rate for every 1-L increase in APC (Figure 2). The *I*^2^ value (90%) indicated high heterogeneity between studies. Furthermore, the funnel plot (eFigure 1 in Supplement 1) suggested evidence of right skew; however, it was unclear whether this is an artifact of the high heterogeneity.^47^ A trim-and-fill analysis was attempted, but it did not result in the removal of any studies. Tests and correction procedures, such as the Egger test and trim-and-fill methods, may also be unreliable or inappropriate in the presence of high heterogeneity.^48,49^

The first sensitivity analysis, excluding 2 studies (3 estimates) that only reported male-specific estimates, demonstrated a 3.43% (95% CI, 2.17%-4.68%) change in the suicide mortality rate for every 1-L increase in APC (eFigures 2 and 3 in Supplement 1), suggesting that their inclusion did not result in a notable difference in the pooled estimate. The sensitivity analysis excluding outliers, specifically a study by Norstrom et al,^34^ resulted in a percentage change of 3.66% (95% CI, 2.44%-4.89%), suggesting that its inclusion also did not result in a notable difference in the pooled estimate (eFigures 4 and 5 in Supplement 1).

The meta-regression model evaluating the impact of geographic location and study year included 11 studies (32 estimates). No evidence was found for the effect of geographic location nor study year (eg, WHO Western Pacific region vs region of the Americas, regression coefficient, −4.95; 95% CI, −15.16 to 5.26; *P* = .34; WHO European region vs region of the Americas, regression coefficient, −0.69; 95% CI, −5.75 to 4.37; *P* = .79; study start date, regression coefficient, 0.07, 95% CI, −0.02 to 0.15; *P* = .13). Nine studies provided sex-specific estimates (27 estimates for male individuals and 24 estimates for female individuals) and were included in a separate meta-regression to evaluate the impact of sex. There was no evidence of sex differences in the respective meta-regression (regression coefficient, −0.10, 95% CI, −1.88 to 1.69; *P* = .91).

### Risk of Bias

Risk of bias was assessed to be high primarily due to concerns related to confounding, as 11 of the 13 studies demonstrated some or high risk of bias within domain 1 due to lack of adjustment for confounders. However, this was likely a consequence of data structure and estimation method. Due to the relatively limited number of data points available, these studies likely lacked the degrees of freedom to accommodate additional covariates in their model. As the introduction of additional covariates may have been inappropriate in this context, we chose not to exclude those studies rated high risk of bias due to their lack of adjustment for confounding.

With respect to other domains, the study by Landberg^35^ raised some concerns related to exposure measurement for Russian estimates, as there may be a large degree of illicit and unrecorded drinking not captured by APC in the given study period. Both the studies by Chen et al^36^ and Skog et al^37^ had some concern with respect to missing data. Both studies relied on single imputation, which has since been superseded by a preference for multiple imputation techniques. However, Skog et al^37^ did conduct additional sensitivity analyses to examine the impact of missing data and accounted for missingness via dummy variables in their model, which may alleviate some concerns related to bias in their study. The study by Testa et al^38^ did not explicitly state whether missing data were present. However, given that this study used cross-sectional data from 2010, there may have simply been no missing data present. The risk of bias results^50^ can be found in eFigure 6 in Supplement 1.

### GRADE Assessment

A summary of the GRADE assessment can be found in the eTable in Supplement 1. All studies included in this review were non–randomized clinical trial studies. Thus, based on GRADE criteria, all studies must begin at a rating of low quality. Concerns regarding risk of bias were the primary reason for down grade. There were minimal concerns regarding inconsistency, indirectness, and imprecision; the degree of publication bias was unclear.

## Discussion

Our study found evidence that, on the population level, every 1-L increase in APC was associated with a 3.59% increase in the suicide mortality rate. The finding of a positive association aligns with previous, albeit mostly outdated, literature reviews.^19,20,21,22^ This finding also aligns with recent meta-analyses by Lange et al,^8,9^ who found that both the average amount of alcohol consumed daily^8^ and alcohol use disorder^9^ were associated with an increased risk of death by suicide. This agreement with individual-level findings suggests that APC, a proxy measure of consumption, could be a useful metric to consider within national suicide prevention strategies.

Our study found no evidence of a sex difference in the association of interest. This result does not align with the recent meta-analysis by Lange and colleagues^8^ on the association between average daily consumption and death by suicide, which suggested that individual-level risk may be elevated for female individuals compared with male individuals. However, the meta-analysis in question^8^ relied on a very limited number of studies (3) for female individuals, and thus the results should be interpreted with caution. The same investigators found that alcohol use disorder was associated with an increased odds of death by suicide, with no statistical difference between the sexes.^9^ Despite not finding sex differences in the current study, we caution that one cannot surmise that policies and interventions targeting alcohol use will have the same net effect for male and female individuals.

Areas of future research include exploring alternative analytical methods, whether the focal association differs between countries, and whether it has changed over time. Although we did not find evidence for regional differences in the association between APC and suicide mortality, the current literature suggests that differences may exist between countries.^39,41,51^ The lack of observed differences in our study may be due to the lack of variation between the included countries, as most were high-income European and North American countries. A large number of existing studies also relied on traditional time series methods or panel data methods for their analyses. While such methods may reduce the risk of finding spurious trend associations,^52,53^ having sufficient power in a time series analysis requires a sufficient number of data points. However, both APC and suicide mortality rates are typically only available annually, which forces studies to rely on fewer data points than what is generally needed. Attempts to increase sample size by using older data may introduce concerns related to data validity and reliability. Thus, alternative analytical methods of longitudinal data analysis, such as mixed-effect models or generalized estimating equations,^54^ both of which may be modified to account for serial autocorrelation in addition to within-participant correlation, should be explored.

The use of the term sex within the current study was done intentionally as suicide mortality is most often ascertained using official statistics or death report data, which document sex as opposed to gender. However, gender is an important and distinct factor that influences both alcohol consumption and suicide mortality. Both the sociocultural perceptions of gender and the impact of oppression and discrimination on gender-diverse populations likely have distinct effects on both alcohol consumption and suicide mortality; however, a lack of data for these groups has made assessing such relationships difficult.^55^ Accordingly, more research is needed to examine the influence of gender on the association between alcohol consumption and suicide mortality.

### Limitations

There are a number of limitations within the present study to acknowledge. First, our overall results relied on a relatively limited number of studies, which were further reduced for the meta-regressions. Second, the current meta-analysis pooled estimates from studies that used an ecological study design; thus, causality cannot be inferred. As with all population-level studies, ecological fallacy remains a notable concern, and one must not attribute population-level results to individuals within a population. Rather, our results demonstrate the usefulness of APC as a population-level metric, that is relatively easy to assess as it is typically measured using economic data, to guide population-level suicide prevention efforts. Our alignment with individual-level findings^10,11^ further highlights the potential of APC to act as a useful proxy. More broadly, ecological studies are still necessary to establish the use of certain population-level metrics in relation to population-level health outcomes, particularly when individual-level metrics demonstrate validity concerns, as is the case with individual-level survey data of alcohol consumption.^14,15,16^ Third, only studies providing estimates that could be transformed into a percentage change were included. As a result, all included studies used somewhat similar estimation methods. It may be the case that our results are an artifact of method choice, and the degree to which model form may influence such results is unclear. Fourth, as discussed previously, most studies did not account for confounding, likely due to concerns with power and overfitting. Thus, the observed association may not reflect reality, although the alignment of our results with the existing literature may alleviate some of our concerns. Fifth, we observed high heterogeneity within our study, which was to be expected given that we pooled estimates both across time and countries. However, it should be noted that *I*^2^ also varies with the sample size of the original studies.^56,57^ Lastly, sex-specific APC was not used within the original studies. Thus, interpretation of sex-specific pooled estimates is related to total consumption rather than a more intuitive sex-specific metric of consumption. It should be noted that in practice, given that APC is ascertained via sales data, valid sex-specific APC is impossible to obtain. Existing sex-specific APC metrics, such as those provided by the WHO Global Information System on Alcohol and Health, represent modeled estimates rather than true APC.^58^

## Conclusions

In this systematic review and meta-analysis, an increase in APC was associated with an increase in the suicide mortality rate on the population level, and this association was similar across the sexes. As such, APC may be a useful target to consider within comprehensive national suicide prevention strategies.

## Notes

Supplement 1. **eAppendix 1.** Example Search in Medline via OVID **eAppendix 2.** Formulas Used for Transformation of Estimates From Their Original Scale to a Percentage-Change Scale **eFigure 1.** Funnel Plot of the Studies on the Association Between APC and Suicide Mortality Included in the Main Meta-Analysis **eFigure 2.** Forest Plot of the Studies on the Association Between APC and Suicide Mortality Included in the Meta-Analysis, Excluding Studies Which Only Reported Male-Specific Estimates (Sensitivity Analysis) **eFigure 3.** Funnel Plot of the Studies on the Association Between APC and Suicide Mortality Included in the Meta-Analysis, Excluding Studies Which Only Reported Male-Specific Estimates (Sensitivity Analysis) **eFigure 4.** Forest Plot of the Studies on the Association Between APC and Suicide Mortality Included in the Meta-Analysis Excluding the Study by Norström et al,^34^ 2012 (Sensitivity Analysis) **eFigure 5.** Funnel Plot of the Studies on the Association Between APC and Suicide Mortality Included in the Meta-Analysis Excluding the Study by Norström et al,^34^ 2012 (Sensitivity Analysis) **eFigure 6.** Risk of Bias in Nonrandomized Studies of Exposure (ROBINS-E) Results **eTable.** Summary of GRADE Assessment

Supplement 2.
Data Sharing Statement
