# Supplementary material for: Alcohol Consumption Per Capita and Suicide: A Meta-Analysis
Source: JAMA Netw Open. 2025 Sep 22;8(9):e2533129. doi: 10.1001/jamanetworkopen.2025.33129 (PMC12455384; doi:10.1001/jamanetworkopen.2025.33129)
Supplement: Supplement 1. — eAppendix 1. Example Search in Medline via OVID eAppendix 2. Formulas Used for Transformation of Estimates From Their Original Scale to a Percentage-Change Scale eFigure 1. Funnel Plot of the Studies on the Association Between APC and Suicide Mortality Included in the Main Meta-Analysis eFigure 2. Forest Plot of the Studies on the Association Between APC and Suicide Mortality Included in the Meta-Analysis, Excluding Studies Which Only Reported Male-Specific Estimates (Sensitivity Analysis) eFigure 3. Funnel Plot of the Studies on the Association Between APC and Suicide Mortality Included in the Meta-Analysis, Excluding Studies Which Only Reported Male-Specific Estimates (Sensitivity Analysis) eFigure 4. Forest Plot of the Studies on the Association Between APC and Suicide Mortality Included in the Meta-Analysis Excluding the Study by Norström et al,34 2012 (Sensitivity Analysis) eFigure 5. Funnel Plot of the Studies on the Association Between APC and Suicide Mortality Included in the Meta-Analysis Excluding the Study by Norström et al,34 2012 (Sensitivity Analysis) eFigure 6. Risk of Bias in Nonrandomized Studies of Exposure (ROBINS-E) Results eTable. Summary of GRADE Assessment [file jamanetwopen-e2533129-s001.pdf]

Supplemental Online Content

Guo K, Jiang H, Shield K, Spithoff S, Lange S. Alcohol consumption per capita and suicide. *JAMA Netw Open.* 2025;8(9):e2533129.
doi:10.1001/jamanetworkopen.2025.33129

eAppendix 1. Example Search in Medline via OVID

eAppendix 2. Formulas Used for Transformation of Estimates From Their Original Scale to a Percentage-Change Scale

eFigure 1. Funnel Plot of the Studies on the Association Between APC and Suicide Mortality Included in the Main Meta-Analysis

eFigure 2. Forest Plot of the Studies on the Association Between APC and Suicide Mortality Included in the Meta-Analysis, Excluding Studies Which Only Reported Male-Specific Estimates (Sensitivity Analysis)

eFigure 3. Funnel Plot of the Studies on the Association Between APC and Suicide Mortality Included in the Meta-Analysis, Excluding Studies Which Only Reported Male-Specific Estimates (Sensitivity Analysis)

eFigure 4. Forest Plot of the Studies on the Association Between APC and Suicide Mortality Included in the Meta-Analysis Excluding the Study by Norström et al,[34] 2012 (Sensitivity Analysis)

eFigure 5. Funnel Plot of the Studies on the Association Between APC and Suicide Mortality Included in the Meta-Analysis Excluding the Study by Norström et al,[34] 2012 (Sensitivity Analysis)

eFigure 6. Risk of Bias in Nonrandomized Studies of Exposure (ROBINS-E) Results

eTable. Summary of GRADE Assessment

This supplemental material has been provided by the authors to give readers additional information about their work.

© 2025 Guo K et al. *JAMA Network Open*.

eAppendix 1. Example Search in Medline via OVID

Ovid MEDLINE: Epub Ahead of Print, In-Process & Other Non-Indexed Citations, Ovid MEDLINE® Daily and Ovid MEDLINE® <1946-Present>

1 alcohol consumption per capita.tw,kf.
2 (alcohol and (per capita or country level or population level)).tw,kf.
3 1 or 2 2055
4 (suicide or suicid* or suicide attempt* or suicide mortality or attempted suicide or death by suicide or self-harm or self injur*).tw,kf.
5 self-injurious behavior/ or self mutilation/ or suicide/ or suicidal ideation/ or suicide prevention/ or suicide, attempted/ or suicide, completed/
6 4 or 5
7 3 and 6

© 2025 Guo K et al. *JAMA Network Open*.

eAppendix 2. Formulas Used for Transformation of Estimates From Their Original Scale to a Percentage-Change Scale

Point estimate (ß): 100*(exp(ß)-1)

Standard Error (SE): 100*SE*exp(ß)

95% Confidence interval: 100*[exp(ß ± 1.96*SE) - 1]

© 2025 Guo K et al. *JAMA Network Open*.

eFigure 1. Funnel Plot of the Studies on the Association Between APC and Suicide Mortality Included in the Main Meta-Analysis

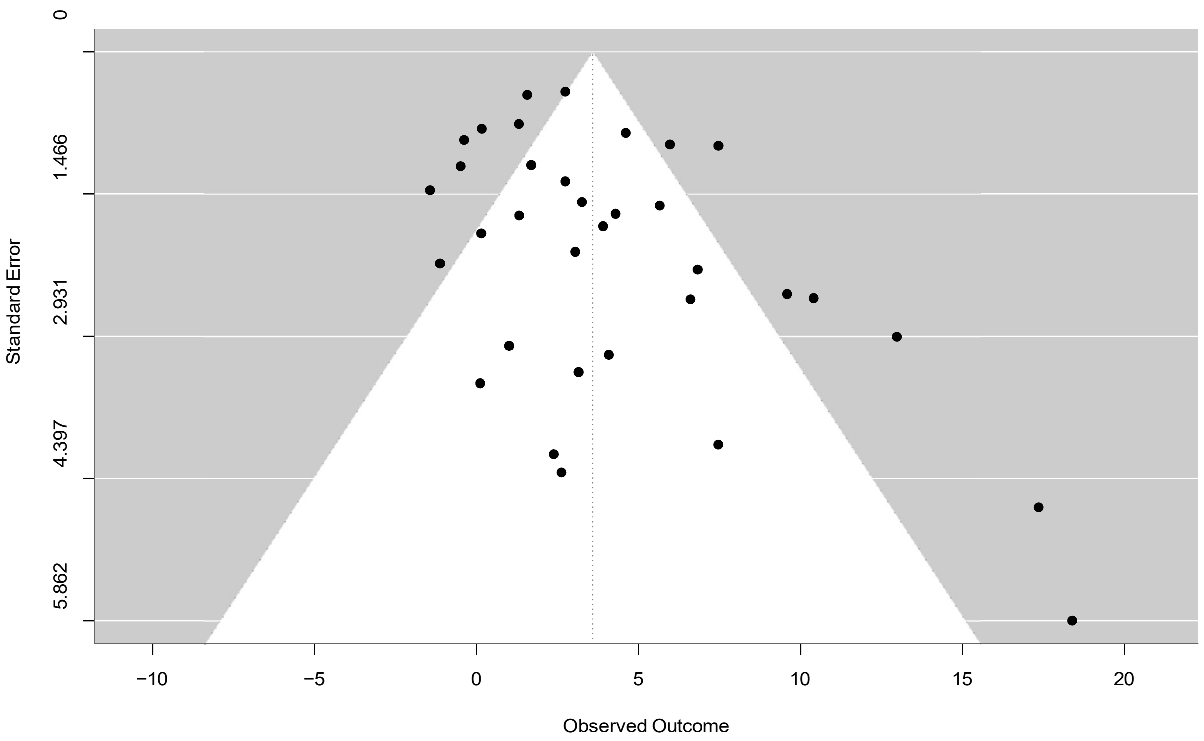

© 2025 Guo K et al. *JAMA Network Open*.

eFigure 2. Forest Plot of the Studies on the Association Between APC and Suicide Mortality Included in the Meta-Analysis, Excluding Studies Which Only Reported Male-Specific Estimates (Sensitivity Analysis)

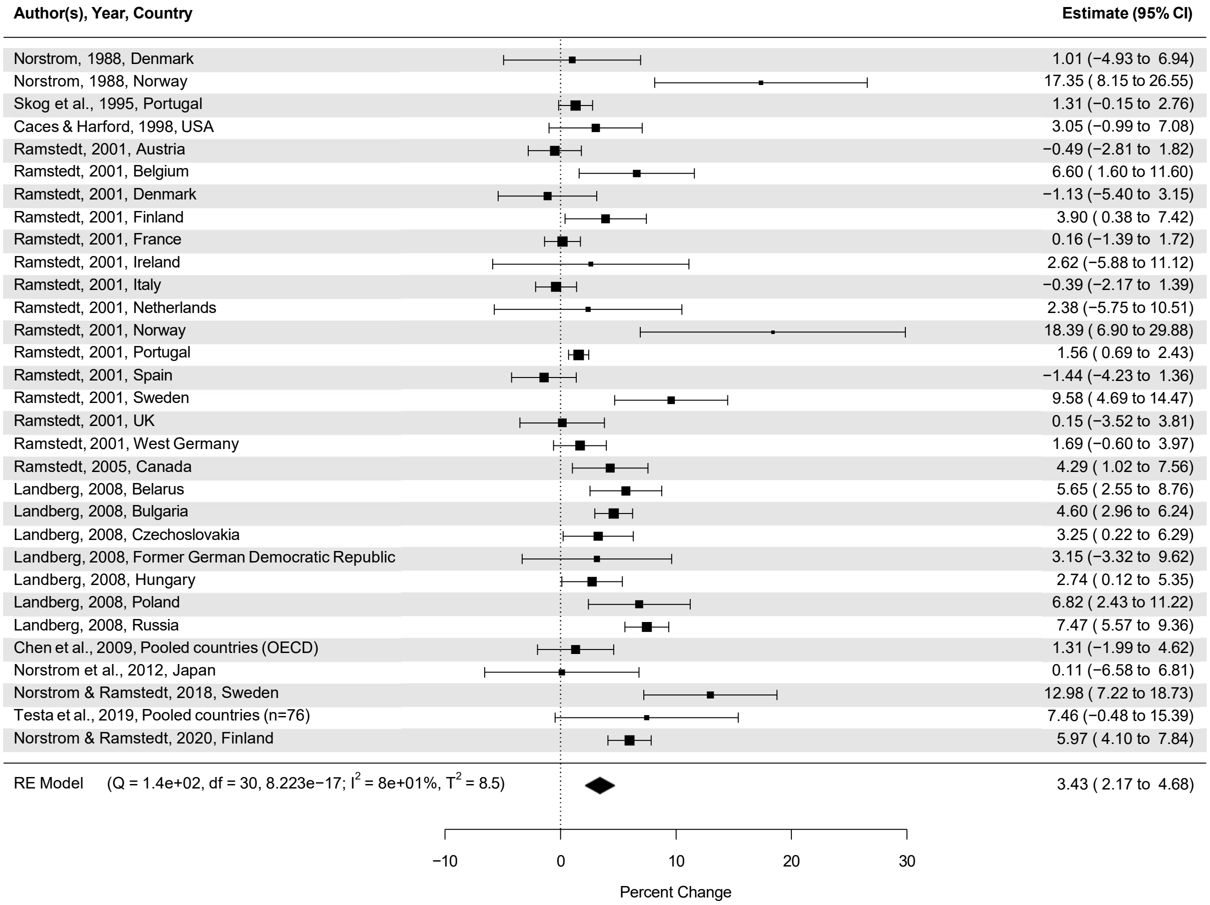

© 2025 Guo K et al. *JAMA Network Open*.

eFigure 3. Funnel Plot of the Studies on the Association Between APC and Suicide Mortality Included in the Meta-Analysis, Excluding Studies Which Only Reported Male-Specific Estimates (Sensitivity Analysis)

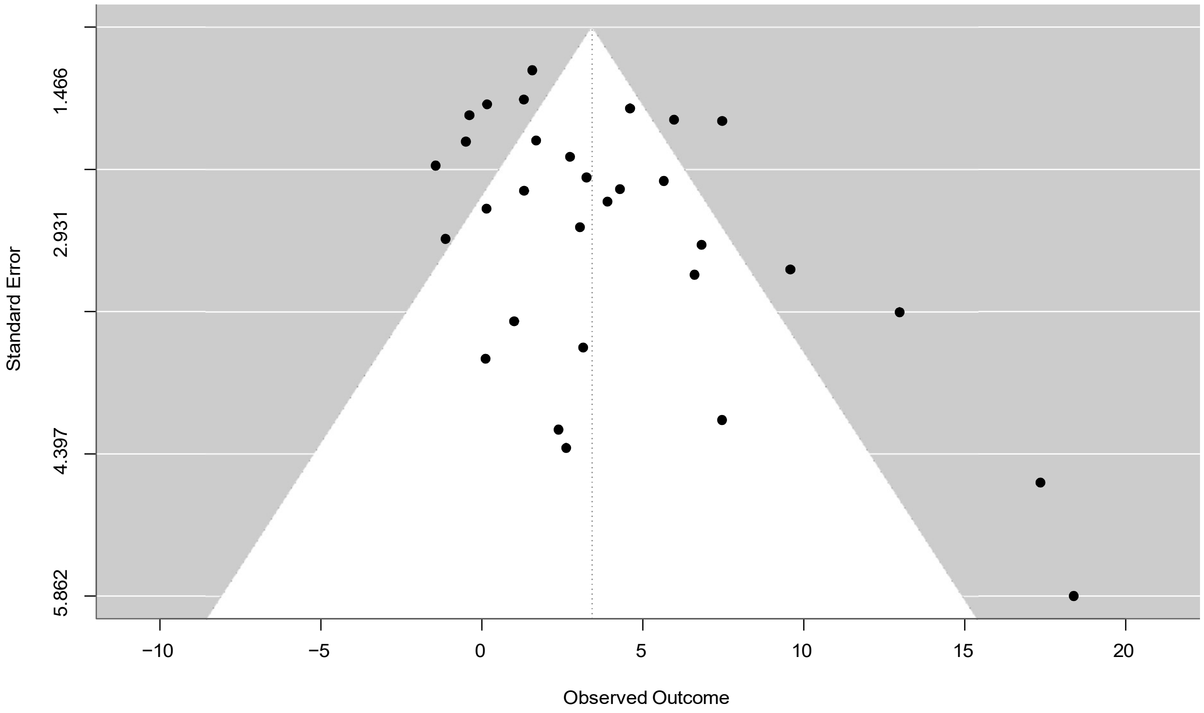

© 2025 Guo K et al. *JAMA Network Open*.

eFigure 4. Forest Plot of the Studies on the Association Between APC and Suicide Mortality Included in the Meta-Analysis Excluding the Study by Norström et al,[34] 2012 (Sensitivity Analysis)

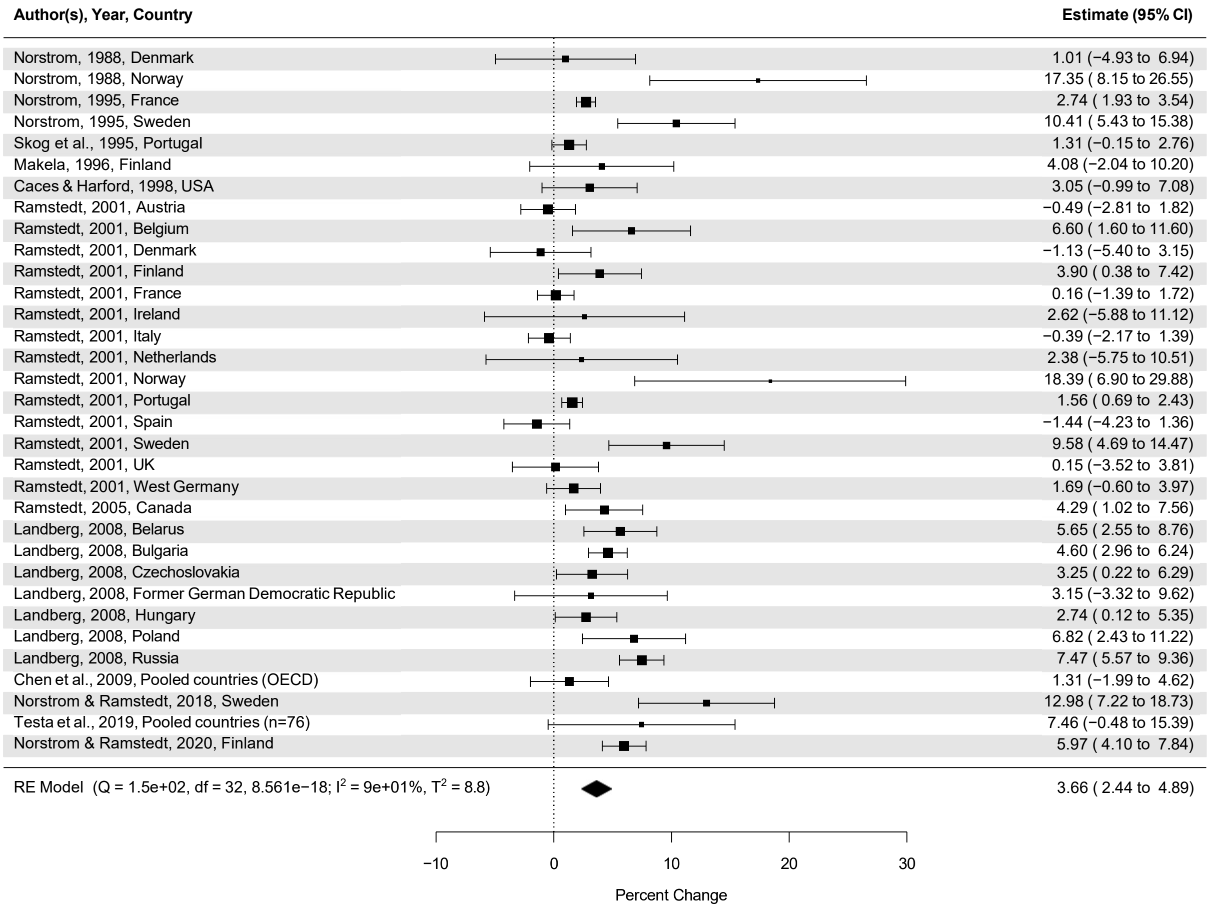

© 2025 Guo K et al. *JAMA Network Open*.

eFigure 5. Funnel Plot of the Studies on the Association Between APC and Suicide Mortality Included in the Meta-Analysis Excluding the Study by Norström et al,[34] 2012 (Sensitivity Analysis)

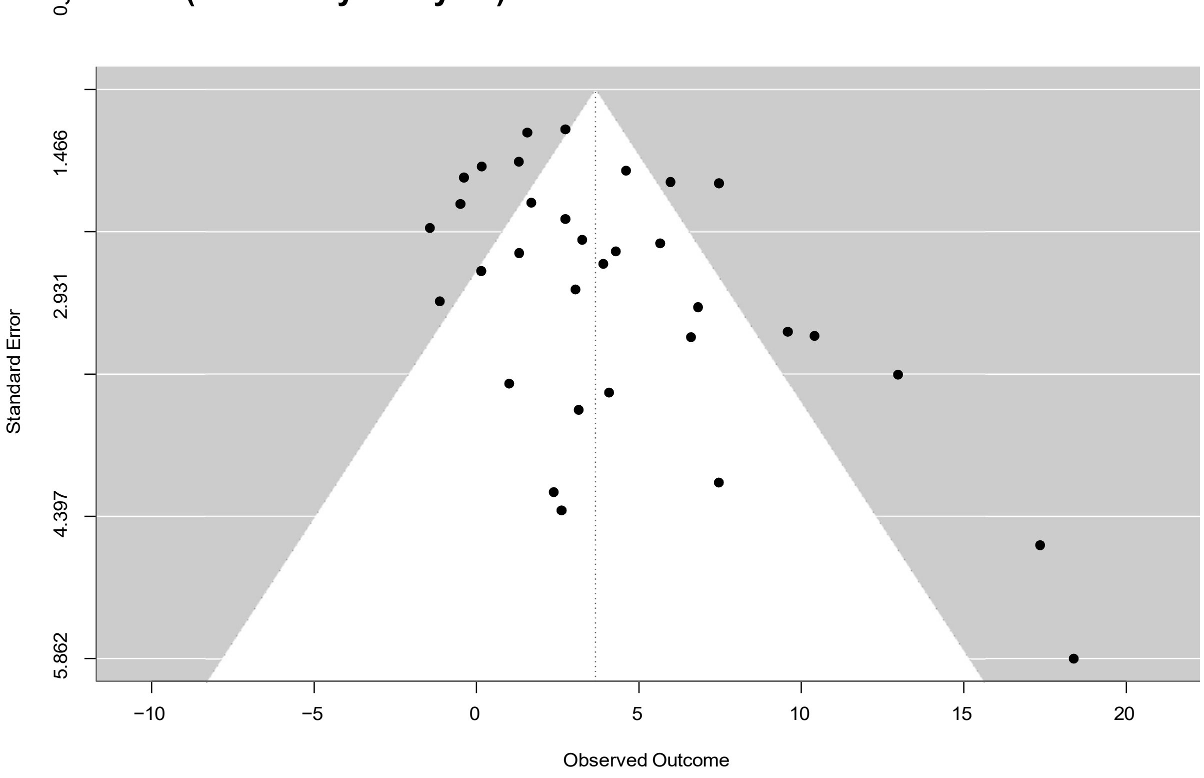

© 2025 Guo K et al. *JAMA Network Open*.

eFigure 6. Risk of Bias in Nonrandomized Studies of Exposure (ROBINS-E) Results

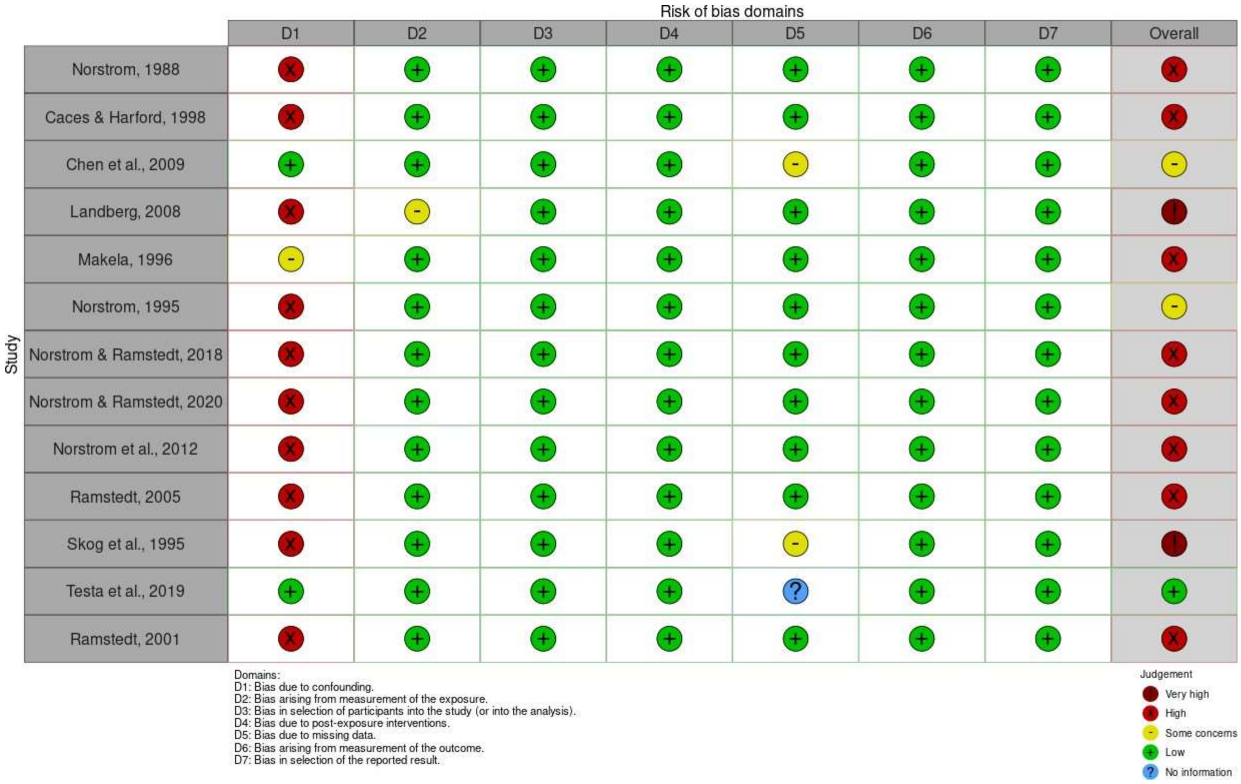

© 2025 Guo K et al. *JAMA Network Open*.

eTable. Summary of GRADE Assessment

Certainty assessment						Effect[a]	Certainty/ Quality	Key Message
Number of studies	Risk of Bias	Inconsistency	Imprecision	Publication Bias	Other considerations			
13	High	Low concern	Low concern	Unclear	NA	3.59	Very low ⊕○○○	Higher APC is associated with higher suicide mortality rates

[a]Percent-change scale

© 2025 Guo K et al. *JAMA Network Open*.
