# Supplementary material for: Alcohol Consumption Per Capita and Suicide: A Meta-Analysis
Source: JAMA Netw Open. 2025 Sep 22;8(9):e2533129. doi: 10.1001/jamanetworkopen.2025.33129 (PMC12455384; doi:10.1001/jamanetworkopen.2025.33129)
Supplement: Supplement 2. — Data Sharing Statement [file jamanetwopen-e2533129-s002.pdf]

Data Sharing Statement

Guo. Alcohol Consumption Per Capita and Suicide. *JAMA Netw Open*. Published September 22, 2025. doi:10.1001/jamanetworkopen.2025.33129

Data
Data available: Yes
Data types: Other (please specify)
Additional Information: Individual study-level data
How to access data: Data will be provided in the publication
When available: With publication

Supporting Documents
Document types: Statistical/analytic code
How to access documents: Statistical/analytical code can be obtained from the corresponding author
When available: With publication

Additional Information
Who can access the data: Anyone requesting the statistical/analytical code
Types of analyses: For any purpose
Mechanisms of data availability: Without investigator support
